# Anticonvulsant effects of aerial parts of *Passiflora incarnata *extract in mice: involvement of benzodiazepine and opioid receptors

**DOI:** 10.1186/1472-6882-7-26

**Published:** 2007-08-08

**Authors:** Marjan Nassiri-Asl, Schwann Shariati-Rad, Farzaneh Zamansoltani

**Affiliations:** 1 Department of Pharmacology, School of Medical Sciences, Qazvin University, Qazvin, Iran; 2 School of Medical Sciences, Qazvin University, Qazvin, Iran; 3 Department of Anatomy, School of Medical Sciences, Qazvin University, Qazvin, Iran

## Abstract

**Background:**

Passion flower (*Passiflora incarnata*) is used in traditional medicine of Europe and South America to treat anxiety, insomnia and seizure. Recently, it has shown antianxiety and sedative effects in human.

**Methods:**

In this study, anticonvulsant effects of hydro- alcoholic extract of Passiflora, Pasipay, were examined by using pentylentetrazole model (PTZ) on mice. Pasipay, diazepam, and normal saline were injected intraperitoneally at the doses 0.4–0.05 mg/kg, 0.5–1 mg/kg and 10 ml/kg respectively 30 minutes before PTZ (90 mg/kg, i.p). The time taken before the onset of clonic convulsions, the duration of colonic convulsions, and the percentage of seizure and mortality protection were recorded. For investigating the mechanism of Pasipay, flumazenil (2 mg/kg, i.p) and naloxone (5 mg/kg, i.p) were also injected 5 minutes before Pasipay.

**Results:**

An ED_50 _value of Pasipay in the PTZ model was 0.23 mg/kg (%95 CL: 0.156, 0.342). Pasipay at the dose of 0.4 mg/kg prolonged the onset time of seizure and decreased the duration of seizures compared to saline group (p < 0.001). At the dose of 0.4 mg/kg, seizure and mortality protection percent were 100%. Flumazenil and naloxone could suppress anticonvulsant effects of Pasipay.

**Conclusion:**

It seems that Pasipay could be useful for treatment absence seizure and these effects may be related to effect of it on GABAergic and opioid systems. More studies are needed in order to investigate its exact mechanism.

## Background

Epilepsy is one of the most common serious neurological conditions. In contemporary society, the frequency and importance of epilepsy can hardly be overstated from the epidemiologic studies. However, in most studies, the overall incidence of epilepsy in developed societies has been found to be around 50 cases per 100,000 persons per year, and rises steeply in older age [1,2]. The current therapeutic treatment of epilepsy with modern antiepileptic drugs (AEDs) is associated with side-effects, dose-related and chronic toxicity, and teratogenic effects, and approximately 30% of the patients continue to have seizures with current AEDs therapy [1,3].

Natural products from folk remedies have contributed significantly in the discovery of modern drugs and can be an alternative source for the discovery of AEDs with novel structures and better safety and efficacy profiles [4]. Now, various phytochemical and pharmacological studies have been carried out on these anticonvulsant plants [5]. Moreover, the number of patients and medical practitioners in the industrialized world which use herbal medicines as a supplement to or substitute for prescription drugs are increased. Herbal medicines are often considered to be a gentle and safe alternative to synthetic drugs. More than half of the medically important pharmaceutical drugs are either natural products or derivatives of natural products [6-8]. In recent years, some experimental studies have indeed evaluated Medieval Iranian medical remedies using modern scientific methods. These studies raised the possibility of revival of traditional treatments on the basis of evidence-based medicine [9].

In Iran, several herbs have been used for anticonvulsant effects from ancient times [10]. In Iran *P. incarnata *is prepared by Iran Darouk Pharmaceutical Co. as the form of tablet and drop with the name of Pasipay and is used in the case of nervous disorder, anxiety, insomnia, muscular tension.

The genus Passiflora consists of 500 species that are mostly found in warm and tropical regions. Passiflora comes from Latin word "Passio" that was first time discovered by Spanish discoverers in 1529 and was described as a symbol for "passion of Christ" [11,12]. This plant was used widely in traditional medicine in West India, Mexico, Netherland, South America, Italia and Argentina for treatment of bronchitis, asthma, whooping cough, pneumonia and insomnia. It also has antianxiety, sedative, antispasmodic and mild anti-microbial effects that are known since long time [12]. One of species of this genus named as *Passiflora incarnata *is more popular than its other species in Europe and in homeopathic medicine; it is used to treat insomnia and anxiety. Passiflora contains several compounds including alkaloids, phenols, glycosyl flavonoids and cyanogenic compounds [12]. In the some experiments, it has potential effects for treatment of some diseases like as anxiety, insomnia, attention- deficit hyperactivity disorder, hypertension and cancer [13-18]. Also, recent study showed that leaves of it had anticonvulsant effects [19].

The effectiveness of Pasipay has been established in treating the physical symptoms of opioid withdrawal in human [20]. But there was no report about the role of opioid system for CNS depressant effects of Passiflora species. Also, there were controversial reports about the role of GABAergic system for CNS effects of it [21,22]. In this study we examined anticonvulsant effects of Pasipay using pentylenetetrazole (PTZ) induced seizure as petit mal epilepsy model in mice. It was predicted that Pasipay would show anticonvulsive effects in PTZ model, which may be due to several mechanism. Thus, we elucidated the possible mechanisms underlying the actions of Pasipay on the CNS and assessed the probable involvement of GABAergic and opioid system.

## Methods

### Animal

Male BALB/c mice (25–30 g) were obtained from the Razi Institute (Karaj, Iran). The animals were individually housed in colony rooms with 12/12 h light/dark cycle at 21 ± 2°C and had free access to food and water. All animal experiments were carried out in accordance with the regulations of the Ethics Committee of the Qazvin University of Medical Sciences.

### Plant material

Hydro- alcoholic extract of Pasipay was obtained from Iran Darouk Pharmaceutical Co. (Tehran, Iran) which was prepared from the standardized extract of leaves, flower and fruit of *P. incarnata*. The total flavonoid content in hydro- alcoholic extracts related to the dried plant material was 4% (w/w) including vitexin and rutin.

### Chemicals

Drugs used as follows: PTZ (Sigma), flumazenil ampoule (2 mg/kg) (Roche), diazepam (Chemidaru, Iran), naloxone (Tolid Daru, Iran). PTZ, diazepam and naloxone were dissolved in normal saline. All compounds were prepared freshly each time and administered intraperitoneally.

### Anticonvulsant activity

#### PTZ-induced seizure

The mice were divided into groups of ten animals each. In the four groups, the mice were given Pasipay at the doses (0.05, 0.1, 0.2, 0.4 mg/kg i.p.) 30 min before the administration of PTZ (90 mg/kg i.p). Two groups were injected diazepam (0.5, 1 mg/kg i.p.) and one group was injected normal saline 30 min before the administration of PTZ (90 mg/kg i.p.) [23]. Each animal is placed into an individual plastic cage for observation lasting 1 h. The onset of a general clonus was used as the endpoint. The general clonus was characterized by forelimb clonus followed by full clonus of the body. The time taken before the onset of clonic convulsions, the duration of clonic convulsions, and the percentage of seizure and mortality protection were recorded [23].

#### The effect of flumazenil on the anticonvulsant activity of Pasipay

We also studied the effects of a selective benzodiazepine receptor antagonist, flumazenil on the anticonvulsant activity of Pasipay in order to investigate the probable involvement of benzodiazepine receptors [24]. It was selected six groups of ten mice each. In the first group, mice were given flumazenil (2 mg/kg) 5 min before the administration of Pasipay (0.4 mg/kg) and 35 min before the injection of PTZ. In the second group, the animals received flumazenil (2 mg/kg) 5 min before the administration of diazepam (0.5 mg/kg). Also, three groups were injected diazepam (0.5 mg/kg i.p.), flumazenil (2 mg/kg) and normal saline 30 min before the administration of PTZ (90 mg/kg i.p.) respectively [23-26]. The anticonvulsant activity of Pasipay and diazepam in mice pretreated with flumazenil was assessed and compared with normal saline (10 ml/kg), flumazenil (2 mg/kg), diazepam (0.5 mg/kg) and Pasipay (0.4 mg/kg) treated animals.

#### The effect of naloxone on the anticonvulsant activity of Pasipay

It was selected four groups of ten mice each for further investigation the probable modulatory activities of opioid receptors on the anticonvulsant activity of Pasipay [27,28]. It was applied naloxone as an opioid receptor antagonist at a dose of (5 mg/kg) 5 min before the administration of Pasipay (0.4 mg/kg) and 35 min before the injection of PTZ in group of ten mice each [23-26]. The anticonvulsant activity of Pasipay in groups pretreated with naloxone was assessed and compared with animals pretreated only with Pasipay (0.4 mg/kg), naloxone (5 mg/kg) and normal saline (10 ml/kg) groups.

### Statistical analysis

The dose of Pasipay to produce an anticonvulsant (ED50) effect in 50 % of animals and its associated 95% confidence limits was calculated by Litchfield and Wilcoxon methods (PHARM/PCS Version 4). The data were expressed as mean values ± S.E.M. and tested with one-way ANOVA followed by the multiple comparison test of Tukey-Kramer. Results with p < 0.05 were taken significant.

## Results

### PTZ-induced seizure

An ED_50 _value of Pasipay in the PTZ model was 0.2 mg/kg (%95 CL: 0.156, 0.342). Pasipay at the dose of 0.4 mg/kg prolonged the onset time of seizure and decreased the duration of seizures compared to saline group (p < 0.001) (Table 1). Pasipay at the dose of 0.2 mg/kg only prolonged the onset time of seizure, compared to saline group (p < 0.001) (Table 1). As it is shown in Table 1, Pasipay exhibited its protection against seizure in a dose-dependent manner. Furthermore, diazepam prolonged the latency and shortened the duration of seizures compared to saline group (Table 1).

**Table 1:** Effects of Pasipay on PTZ-induced convulsion in mice

**Treatment (dose)**	**Onset (sec)**	**Duration (sec)**	**Seizure protection (%)**	**Mortality protection (%)**
Normal saline (10 ml/kg)	51.83 ± 64	12 ± 1.80	0	0
Diazepam (0.5 mg/kg)	485.5 ± 74.97***	3.5 ± 2.21*	80	90
Diazepam (1 mg/kg)	600 ± 0***	0 ± 0***	100	100
Pasipay (0.05 mg/kg)	56 ± 4.51	15.3 ± 4.48	0	0
Pasipay (0.1 mg/kg)	112.66 ± 16.2	7.1 ± 2.5	10	50
Pasipay (0.2 mg/kg)	137.6 ± 17.8***	7 ± 1.1	20	80
Pasipay (0.4 mg/kg)	600 ± 0***	0 ± 0***	100	100

Normal saline, diazepam and Pasipay were administered i.p. 30 min before the injection of PTZ (90 mg/kg, i.p.); Values are the mean ± S.E.M. for 10 mice. *p < 0.05;*** p < 0.001, compared to saline group, Tukey-Kramer test.

### The effect of flumazenil on the anticonvulsant activity of Pasipay

In the PTZ-induced seizure model, the administration of flumazenil (2 mg/kg) 5 min before Pasipay (0.4 mg/kg) reversed the effect of Pasipay in prolonging seizure latency and reducing the duration of clonic seizures. There was no significant difference between the latency and duration of seizure in mice which received Pasipay (0.4 mg/kg) pretreated with flumazenil and the saline group. Also, flumazenil could reverse the anticonvulsant activity of diazepam (Table 2).

**Table 2:** Effect of flumazenil on the anticonvulsant activity of Pasipay and diazepam in PTZ-induced convulsion in mice

**Treatment (dose)**	**Onset (sec)**	**Duration (sec)**	**Mortality protection (%)**
Normal saline (10 ml/kg)	51.83 ± 64	12 ± 1.80	0
Flumazenil (2 mg/kg)	44.71 ± 1.44	8.25	85
Diazepam (0.5 mg/kg)	485.5 ± 74.97***	3.5 ± 2.21*	90
Diazepam+ Flumazenil	152.5 ± 28.9	12.25 ± 3.42	70
Pasipay (0.4 mg/kg)	600 ± 0***	0 ± 0***	100
Pasipay+ Flumazenil	184.33 ± 29.1	11 ± 1.52	50

Normal saline, diazepam and Pasipay were administered (i.p.) 30 min before the PTZ (90 mg/kg, i.p.); Flumazenil was administered 35 min before the injection of PTZ (90 mg/kg, i.p.); Values are the mean ± S.E.M. for 10 mice; *p < 0.05; ***p < 0.001, compared with saline group, Tukey-Kramer test.

### The effects of naloxone on the anticonvulsant activity of Pasipay

Pretreatment of mice with naloxone (5 mg/kg) 5 min before the administration of the Pasipay (0.4 mg/kg) reversed the reduction in seizure duration. However, the time course of the seizure threshold in mice was not reversed completely by naloxone and it was significant compared to control (p < 0.001) (Table 3).

**Table 3:** Effect of naloxone on the anticonvulsant activity of Pasipay in PTZ-induced convulsion in mice

**Treatment (dose)**	**Onset (sec)**	**Duration (sec)**	**Mortality protection (%)**
Normal saline (10 ml/kg)	51.83 ± 64	12 ± 1.80	0
Naloxone (5 mg/kg)	10.66 ± 0.66	49.6 ± 4.34	0
Pasipay (0.4 mg/kg)	600 ± 0***	0 ± 0***	100
Pasipay+ Naloxone	125.4 ± 2.87***	8.81 ± 2.64	40

Normal saline and Pasipay were administered (i.p.) 30 min before the injection of PTZ (90 mg/kg, i.p.); Naloxone was administered 35 min before the injection of PTZ (90 mg/kg, i.p.); Values are the mean ± S.E.M. for 10 mice; *** p < 0.001, compared to saline group, Tukey-Kramer test.

## Discussion

The present study investigated the anticonvulsant effect of Pasipay using the PTZ-model. Pasipay could suppress onset and duration of clonic seizure in PTZ model and it seems that this effect increased dose dependently. Also seizure and mortality protection percent increased dose dependently as we could observe that at the dose of 0.4 mg/kg, all animals were protected against seizure and mortality significantly and this effect was similar to diazepam 1 mg/kg.

This study is in agreement with a recent report by Dhawan et al, however, we have seen anticonvulsant effects of extract at the lower doses. This could be explained by several reasons: Our extract was the standard hydroalcoholic extract of aerial parts of herb which was prepared as the drug formulation, Pasipay, by Iran Darouk Pharmaceutical Co. But, the previous work was the methanolic extract of the leaves of *Passiflora incarnata *[19]. Moreover, there are several controversial reports about the CNS effects of *P. incarnata *extracts and their active component which could be related to different active component of it [29-33]. Meanwhile, in our study, the major flavonoids of Pasipay were also different from previous studies. In addition, there is a different between the sources of *P. incarana *of our work and previous work.

Clonic seizure was induced by γ-aminobutyric acid (GABA) transmission blocker PTZ [34]. Regarding the possible contribution of GABAergic system in the anticonvulsant activity of Pasipay, flumazenil, a benzodiazepine receptor antagonist, was used [24]. As it was shown in table 2, flumazenil decreased the prolongation of seizure latency induced by Pasipay and it also antagonized the effect of Pasipay on decreasing the duration of clonic seizures in the PTZ model. It is noteworthy that the anticonvulsant effect of Pasipay is blocked by an antagonist of benzodiazepine receptor. So this effect of Pasipay seems to be related to benzodiazepine receptor activation. This result is similar to previous finding by Fernandez et al [21]. They found that anxiolytic effects of one component of Passiflora were related to benzodiazepine receptors activation. However, there is a controversial study which reported that anxiolytic effects of *P. incarnata *extract were not mediated through an action on the benzodiazepine/GABA receptors [22]. On the other hand, it is found that many flavonoids could act as benzodiazepine- like molecules in the central nervous system (CNS) and modulate GABA-generated chloride currents in animal models of anxiety, sedation and convulsion [21]. It is possible that anticonvulsant activities of Pasipay related to its flavonoids like rutin and vitexin as was determined in the Pasipay. Recently, rutin had sedative and sleep- enhancing effects in mice [21]. However, there is a controversial study which reported that pure vitexin and isovitexin of *P. incarnata *had no activity in CNS tests [35]. Further studies need to make clear which of these flavonoids or other compounds have anticonvulsant effects.

We also found other mechanism about the anticonvulsant effects of Pasipay. As it was shown in table 3, naloxone only antagonized the effect of Pasipay on decreasing the duration of clonic seizures in the PTZ model compared to saline group. Naloxone decreased the prolongation of seizure latency induced by Pasipay. However, it did not show any significant reversal of Pasipay effects. It seems that some part of anticonvulsant effects of it related to activation of opioid system which was attenuated by naloxone. Since, one study reported that concurrent co-administration of benzoflavon moiety of *P. incarnata *with morphine attenuated naloxone-precipitated withdrawal jumps [12]. Thus, we used naloxone as a non-specific opioid receptor antagonist for preliminary study to clear the exact mechanism of this herb.

On the other hand, anticonvulsant activity of kappa opioid receptor (KOPr) agonists has been established in wide range of previous animal studies. KOPr agonists are effective against bicuculline-, maximal electroshock- and excitatory amino acid-induced convulsions. Furthermore, they attenuate the kindling of seizures produced by repeated administration of PTZ [36-39]. Furthermore, dynorphine, an endogenous opioid peptide, binds to KOPr. It has anticonvulsant effects in previous studies [28]. There is one hypothesis that Pasipay could active KOPr and produce protective effects against PTZ-induced seizure. However, the mechanism of anticonvulsant effects with KOPr agonist, have not been universal. Modulatory effects of its agonist on seizure induced by GABA_A _receptor antagonists were reported [36]. Furthermore, its agonist could inhibit glutamate release [40,41]. Thus, there are two possibilities which could explain the anticonvulsant activity of the Pasipay via the KOPr activation: 1) enhancement GABAergic activity or 2) attenuation glutamatergic activity.

## Conclusion

In brief, the present study provides evidence for anticonvulsant activity of Pasipay in the clonic seizure of PTZ model. As the protective effects of Pasipay in clonic seizure it suggests that it could be useful for treatment of absence seizure. Furthermore, the important role of benzodiazepine receptor in the effects of Pasipay should be considered.

Also, the opioid receptor mechanism is apparently involved in the response induced by Pasipay which should be investigated.

## Competing interests

The author(s) declare that they have no competing interests.

## Authors' contributions

MNA is the primary author and wrote the manuscript, participated in the design of the study and performed the statistical analysis and revised the manuscript. SSR and FZ helped in the design of the study and wrote the manuscript. All of the authors have read and approved the final manuscript.

## Pre-publication history

The pre-publication history for this paper can be accessed here:


